# Do Refuge Plants Favour Natural Pest Control in Maize Crops?

**DOI:** 10.3390/insects8030071

**Published:** 2017-07-18

**Authors:** Reinaldo Quispe, Marina Mazón, Alexander Rodríguez-Berrío

**Affiliations:** 1 Fundación PROINPA, Regional Altiplano, La Paz, Bolivia; 2 Biodiversity and Ecosystem Services Research Program, Universidad Nacional de Loja, Ciudadela Universitaria, sector La Argelia, EC 110101 Loja, Ecuador; 3 Departamento de Ciencias Ambientales y Recursos Naturales/Instituto de Investigación de Biodiversidad CIBIO, Universidad de Alicante, Apdo. Correos 99, 03080 Alicante, Spain; 4 Departamento Académico de Entomología, Facultad de Agronomía, Universidad Nacional Agraria La Molina, Av. La Molina s/n, Distrito La Molina, Lima 12, Peru

**Keywords:** refuge plant, functional groups, predators, parasitoids, maize crop

## Abstract

The use of non-crop plants to provide the resources that herbivorous crop pests’ natural enemies need is being increasingly incorporated into integrated pest management programs. We evaluated insect functional groups found on three refuges consisting of five different plant species each, planted next to a maize crop in Lima, Peru, to investigate which refuge favoured natural control of herbivores considered as pests of maize in Peru, and which refuge plant traits were more attractive to those desirable enemies. Insects occurring in all the plants, including the maize crop itself, were sampled weekly during the crop growing cycle, from February to June 2011. All individuals collected were identified and classified into three functional groups: herbivores, parasitoids, and predators. Refuges were compared based on their effectiveness in enhancing the populations of predator and parasitoid insects of the crop enemies. Refuges A and B were the most effective, showing the highest richness and abundance of both predators and parasitoids, including several insect species that are reported to attack the main insect pests of maize (*Spodoptera frugiperda* and *Rhopalosiphum maidis*), as well as other species that serve as alternative hosts of these natural enemies.

## 1. Introduction

The use of non-crop plants to provide resources that pests’ natural enemies need, such as alternative prey, refuge, or additional food [1], is being increasingly incorporated into integrated pest management programs [2]. The most effective natural enemies of phytophagous insects are entomophagous insects, i.e., predators and parasitoids. These natural enemies are more specialized than other biological control agents such as entomopathogenic fungi, since the host range they attack are usually rather narrow, especially parasitoids, so that they influence the community structure of herbivorous insects more than other unspecialized enemies [3]. Both functional groups have shown higher fecundity and longevity rates when feeding on other resources like nectar (floral and extrafloral) or pollen [1,4,5,6], which are not usually available in agricultural systems. Planting vegetation contiguous to the crops may provide those resources, as well as refuge areas when the crops used are annuals, not perennials, e.g., cereals, sugarcane, or maize. Natural enemies have been shown to move from edge vegetation to the centre of a standing crop, thus enhancing natural control [7]. However, not all flowering plants are suitable to favour these natural enemies. Nectar should be easily available for predators and parasitoids, which usually have short mouthparts that limit their access to narrow or deep corollas [8,9]. Furthermore, not all natural enemies attracted by those plants equally favour natural pest control in all crops. Surveys at species-level (or at least genus-level) must be carried out in order to know which species are mostly favoured and if they have any impact on herbivore suppression.

Maize (*Zea mays* L.) is one of the most important annual crops grown in Peru, where its cultivation and consumption date ca. 3000 years BP [10]. However, many insect pests reduce its potential yield, such as the aphid *Rhopalosiphum maidis* (Fitch) [11], although *Spodoptera frugiperda* (J. E. Smith) is by far the most important in most part of the neotropics [12]. Some parasitoids have been recorded attacking *S. frugiperda* in South America, such as *Campoletis grioti* (Blanchard), *Cotesia margiventris* (Cresson), *Chelonus insularis* (Cresson), *Apanteles* spp and several species of Tachinidae [13,14,15], as well as several predators like Reduviidae (Hemiptera), Coccinellidae (Coleoptera), and larvae of Syrphidae (Diptera) [16]. However, farmers are still reluctant to incorporate agroecological practices, and very large amounts of pesticides are being applied to maize crops. There is an urgent need to improve the efficiency of different agroecological methodologies to control maize pests.

The aim of the present work was (1) to conduct a survey on which plant refuges might effectively favour natural pest control of maize crops in Peru, and (2) to identify which plant attributes are of most benefit to natural enemies of herbivorous insect crop pests.

## 2. Materials and Methods

### 2.1. Study Area

The study took place in Fundo La Molina, an experimental farm of the Universidad Nacional Agraria La Molina (UNALM), Lima (Peru), with coordinates 12°05′06″ S, 76°57′09″ W, 251 meters above the sea level.

This region is classified as a subtropical desert [17], placed in the Pacific Desert ecoregion [18], with mean annual rainfall lower than 30 mm, under warm-temperate climate conditions, with warm summers (December–March) of 22–30 °C and temperate winters (June–September) of 13–22 °C.

The experiment was carried out next to a 15,000 m^2^ maize plot of PM-303 variety. This is a commercial crop with non-ecological management, with several pesticides being applied during the experiment: metomil (carbamate, labelled as highly dangerous), clorpirifos (organophosphate, labelled as moderately dangerous) and lambdacihalotrina (pyrethroids, labelled as slightly dangerous). Maize was sown on 10 January and harvested on 6 June 2011.

### 2.2. Field Experiments Setup

Based on literature on plants that attracted beneficial insects [1,2,19], we selected 15 flowering plant species, distributed randomly into three refuge patches (Table 1).

**Figure 1:**
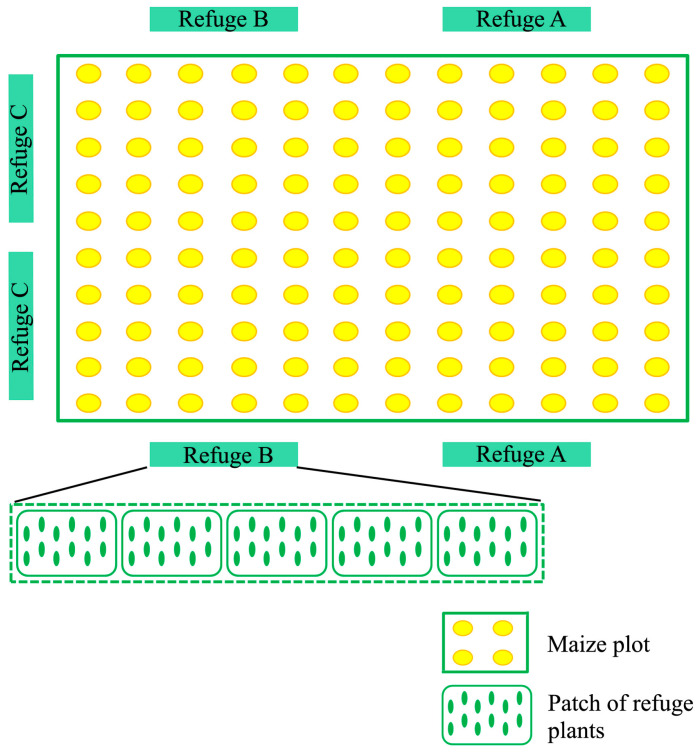
Sampling design of the field experiment.

Approximately 30 seeds of every species were sown in the plant nursery of UNALM in November 2010. One week before the maize sowing they were transplanted in the field, when seedlings had seed leaves, in such a way as to create five 7.5 m^2^ plots or “refuge patches”, made up of 10–12 seedlings of one species each. The three refuge patches were replicated once, and all of them were established in close proximity to the maize plot, as shown in Figure 1.

### 2.3. Insect Sampling and Identification

Sampling in both the refuges and the maize plot took place weekly during the crop growing cycle, from February to June 2011.

In every refuge all plants were sampled by hand for 15 minutes, with all adult insects observed in association with the plant being collected. Three additional samples were made using a sweeps net for every refuge plant. In the maize plot, 50 random plants were sampled following the methodology proposed by Sánchez and Sarmiento [20], i.e., a zigzag sampling starting at 10 m from the maize plot border and at tenth furrow, with the collection of all adult insects present on the plant.

Sampling was done from 9:00 to 15:00 h, and all insects were preserved in 70% ethanol for identification.

All specimens were identified to species or sorted into morphospecies, and classified into functional groups, amongst which only three of them were considered in this study: herbivores, parasitoids, and predators.

Materials studied were deposited at the Museum of Entomology “Klaus Raven Buller” in UNALM.

### 2.4. Data Analysis

The effectiveness of the sampling effort was evaluated by richness estimators, which predict the potential total species richness of the refuges and the maize plot. Effectiveness is given as the proportion of species collected in relation to the richness predicted by estimators ICE (incidence-based coverage estimator) and Jacknife 1 [21].

Suitability of plant species as favourable refuges for natural pest control was given by two aspects: to harbour parasitoids and predators also present in maize crops, and to provide alternative food sources for those natural enemies. We compared insect species composition of every functional group occurring in all the plants and the maize crops by using the Jaccard index, which measures similarities of species assemblages by their presence or absence. Data obtained were then represented in a multidimensional scale analysis (MDS), where all samples are situated in a 2D plot with distances representing similarities in the species composition of samples.

In order to determine which plant attributes were related most closely with species richness (number of species) and abundance (number of individuals) of parasitoids and predators, a Kruskal-Wallis test was carried out separately for every refuge, considering all samples for every plant species as replicates. Relationship amongst plant attributes and species diversity (measured by the Shannon-Wiener index) of parasitoids and predators was assessed by a randomization test with 1000 random partitions [22]. The plant attributes evaluated were: extrafloral nectaries (presence/absence), foliage density (high/medium), and life form (herb/shrub/woody). Furthermore, a Pearson correlation coefficient (R) was calculated to estimate relationships between the parasitoid and predator variables described above and the diversity, richness, and abundance of herbivores in every refuge.

Alpha-diversity indices and randomization tests were calculated and performed with the software Species Diversity and Richness 3.02 (Pisces Conservation Ltd., Pennington, Lymington, UK). Both richness estimations and beta-diversity index were obtained with StimateSWin8.2.0 [23]. MDS, Kruskal-Wallis, and correlation analyses were performed by means of the software Statitstica 7.0 (Stat Soft, Inc., Tulsa, OK, USA).

## 3. Results

A total of 12,459 individuals were collected, corresponding to 179 species, from which 4114 individuals were collected in the maize crop and the remaining 9345 in the refuge areas, being Refuge A, where the highest abundance and species richness were found (Table 2). Herbivores and predators were the most abundant functional groups, while parasitoids were, by far, the richest group in all refuges, providing nearly half the total species recorded in the whole sampling. The plant species *Foeniculum vulgare* and *Gossypium barbadense* in Refuge A, *Bidens pilosa* in Refuge B, and *Malva parviflora* and *Galinsoga parviflora* in Refuge C, retained the highest number of both parasitoid and predator species.

Sampling effort in Refuge B was the most effective, having collected more than 90% of potential richness according to ICE, whilst in Refuge C sampling reached around 75% of potential richness (Table 3). Considering sampling in each plant species, there was a high variability in effectiveness, with values from 29.61% to 86.58% of potential total richness in *Artemisia absinthium* and in maize crops, respectively (Table 3). The two estimators gave very different predictions for several plant species, with ICE usually overestimating species richness. The plant species with the most similar and highest values for sampling effectiveness were *F. vulgare* (81.71 and 80.74% for ICE and Jacknife 1, respectively) and *G. barbadense* (74.82 and 74.71%) in Refuge A, *B. pilosa* (81.06 and 77.98%) and *H. annus* (64.15 and 70.86%) in Refuge B, and *M. parviflora* (67.57 and 72.70%) and *P. vulgaris* (63.33 and 73.46%) in Refuge C.

**Figure 2:**
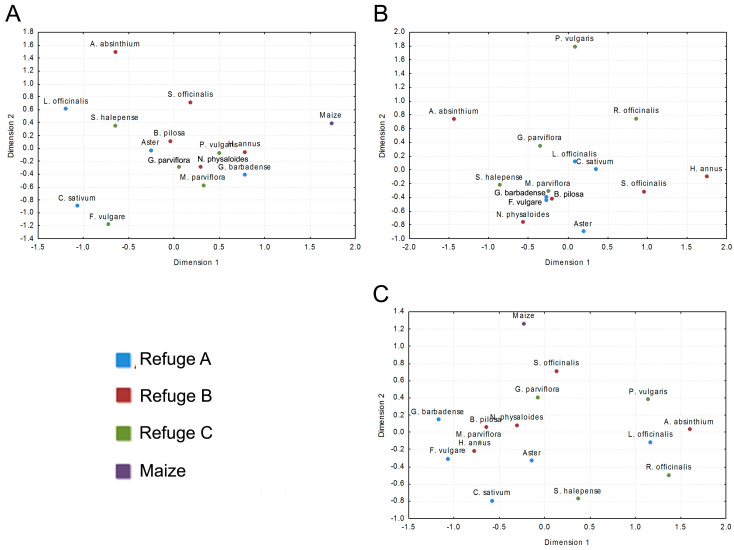
Multidimensional scale (MDS) plot from the Jaccard index similarities matrix amongst insect compositions occurring in every plant species from each refuge, for each functional group: (**A**) herbivores, (**B**) parasitoids, and (**C**) predators.

Insect assemblages found in refuge plants are rather dissimilar to those found in the maize plot, and similarities amongst them seem to be independent from the refuges where plants were sown (Figure 2). Regarding functional groups, herbivore and predator assemblages seemed to be somewhat different in all plants (Figure 2A,C). However, *G. barbadense*, *F. vulgare* (Refuge A), *B. pilosa* (Refuge B), and *M. parviflora* (Refuge C), despite being situated far from each other (except *F. vulgare* and *G. barbadense*), all shared a high proportion of parasitoid species, having very closely related assemblages (Figure 2b).

**Figure 3:**
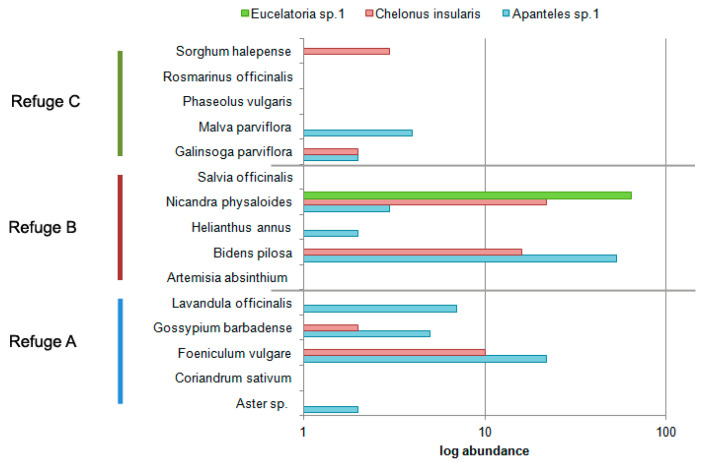
Log abundance of the three parasitoid species reported as enemies of *Spodoptera frugiperda*, in every refuge plant from each refuge.

**Figure 4:**
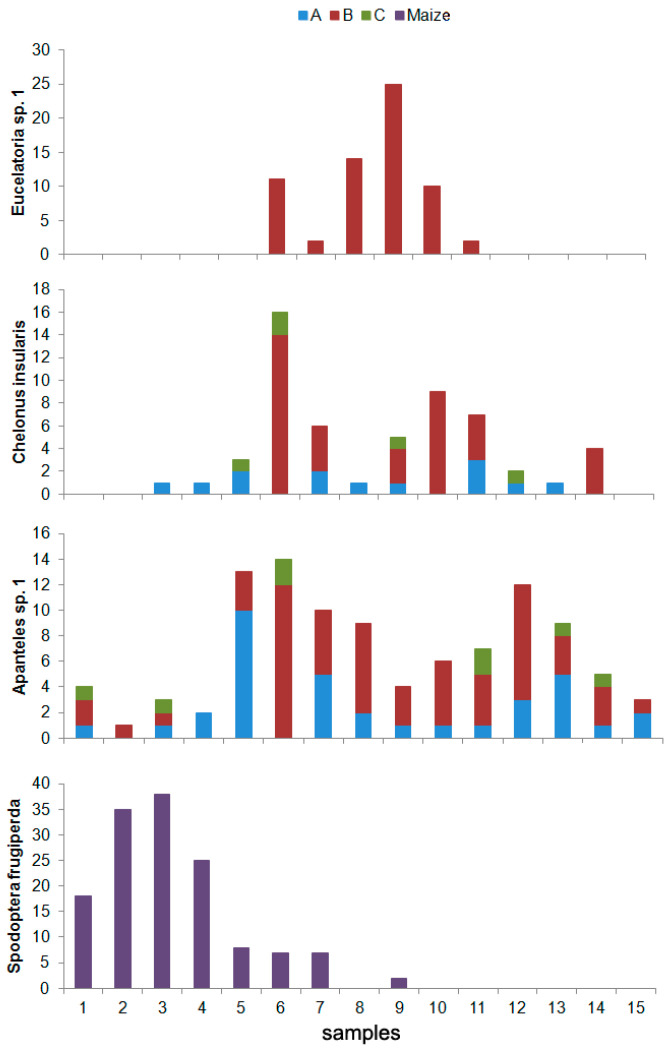
Abundance of *Spodoptera frugiperda* and the three parasitoid species reported as its enemies during the sampling period in each refuge.

From the 15 herbivore species found in the maize crop plants, only six species were also recorded in the refuges, but none of them was found in *F. vulgare* (Refuge A), *A. absinthium* (Refuge B), or *R. officinalis* (Refuge C) (Table S1). Among parasitoids, not a single species was present both in maize crops and in the refuges (Table S1). However, amongst all parasitoids collected in the refuges, there are three species that have been reported as parasitoids of *Spodoptera frugiperda*: *Eucelatoria* sp. 1, *Chelonus insularis*, and *Apanteles* sp. 1, which were more abundant in Refuge B, especially in *N. physaloides* and *B. pilosa*, although in *P. vulgare* (refuge A) there was a high abundance of *Apanteles* sp. 1 and *C. insularis* (Figure 3). The abundance of these parasitoids along the sampling period is asynchronous to the phenology showed by *S. frugiperda* (Figure 4). However, the most abundant parasitoid in the whole sampling was *Praon volucre* (Haliday), only present in Refuge B (specifically in *H. annus*) (Table S1).

**Figure 5:**
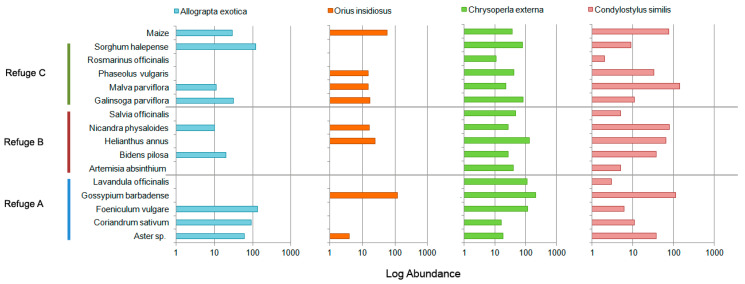
Log abundance of the four main predators occurring in maize, in every refuge plant.

**Figure 6:**
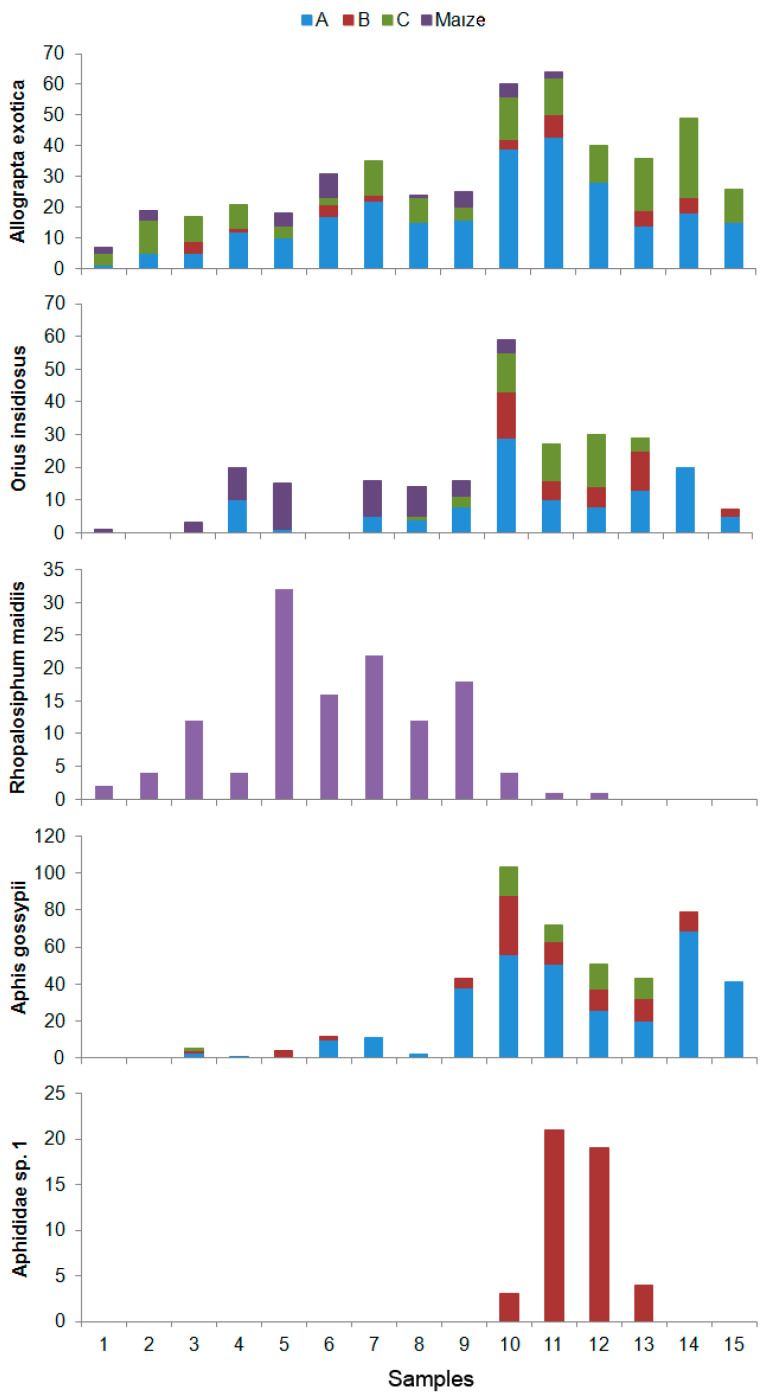
Abundance of two of the four main predator species occurring in maize (*Allograpta exotica* and *Orius insidiosus*), and three of their main preys (*Rhopalosiphum maidis*, *Aphis gossypii*, and *Aphididae* sp. 1) during the sampling period in each refuge.

Half of the predator species found in maize crops were also collected from all of the refuges (Table S1), amongst which four species were the most abundant: *Allograpta exotica*, *Orius insidiosus*, *Chrysoperla externa*, and *Condylostylus similis*. Adults of *C. externa* were not the most abundant predators in maize, although it is very likely that the Chrysopidae nymphs that were found on this crop belonged to that species. *A. exotica* was mainly found in Refuges A and C, especially in *Foeniculum vulgare* and *S. halepense*, respectively; *O. insidiousus* was mainly found in Refuge C, although its highest abundance was recorded in *G. barbadense* (refuge A); *C. externa* and *C. similis* were abundant in the three refuges (Figure 5). *Chrysoperla* and *Consdylostylus* are more generalist, whilst *Orius* and *Allograpta* feed mostly on aphids, and they were both absent in the last samples recorded in maize (Figure 6). Only one species of aphid was found in maize (*Rhopalosiphum maidis*), whose activity period coincides with records of those predator species in maize; however, two additional aphid species were collected in the refuges (*Aphis gossypii* and an unidentified species), which were present in the last samples made during the sampling period (Figure 6).

When plant attributes were tested, only the presence of bracteal extrafloral nectaries showed consistent and significant results in all of the refuges, favouring both parasitoids and predators (Table 4); in Refuge C, where extrafloral nectaries were not in bracts but in the stipules (specifically in *Phaseolus vulgaris*), predators abundance or richness was not affected. The randomization tests performed for diversity indices also supported the positive relationship (*p* < 0.05) of the presence of bracteal extrafloral nectaries to these two functional groups.

A higher foliage density favoured both parasitoids and predators in Refuge A, and also parasitoids in Refuge C (Table 4). Regarding the life form, parasitoids are positively more related to shrubs in Refuge B, whilst predators seem to consistently prefer herbs. When randomization tests were performed for diversity indices, only diversity of predators was found to be significantly related (*p* < 0.05) to foliage density, being largest when density is high, occurring in all refuges. No significant results were obtained for life form and insect diversity.

Abundance and richness of predators was positively and significantly (*p* < 0.05) correlated in all the refuges, to both abundance (R = 0.644 and 0.654 for abundance and richness of predators, respectively, in Refuge A; R = 0.484 and 0.453 in Refuge B; R = 0.329 and 0.311 in Refuge C) and richness of herbivores (R = 0.511 and 0.537 for abundance and richness of predators, respectively, in Refuge A; R = 0.485 and 0.485 in Refuge B; R = 0.314 and 0.299 in Refuge C). Parasitoid abundance and richness were only significantly correlated to abundance and richness of herbivores in Refuge A (R = 0.345 and 0.331 for abundance and richness of parasitoids, respectively, correlated to herbivores abundance; R = 0.248 and 0.236, respectively, correlated to herbivores richness), and only parasitoid abundance was correlated to herbivores abundance in Refuge B (R = 0.186).

## 4. Discussion

In the present study, we assessed the efficiency of three refuges consisting of five different plant species known to favour natural enemy control of herbivorous insect pests found in maize. This efficiency was considered as the best enhancement of pests’ natural enemies, i.e., predators and parasitoids, focusing on those attacking maize herbivores. This enhancement is understood as having the potential for maintaining and increasing a long-term population of these natural enemies which, otherwise, are not able to survive in a perennial crop. Bearing this in mind, the most suitable refuges of those assessed were Refuges A and B. Refuge A retained most of the main predator species that were also present in the maize plot, whilst in Refuge B the largest abundance of parasitoids reported as enemies of *S. frugiperda* was found. In Refuge A, *Foeniculum vulgare* and *Gossypium barbadense* had very similar parasitoid assemblages, showing the highest parasitoid and predator richness amongst the species sown in that refuge. In these two species, parasitoid Hymenoptera were found more frequently by other authors [19,24,25], and they are the only ones in Refuge A having extrafloral nectaries in their bracts [26], a trait that significantly favoured parasitoids and predators in all refuges, both in terms of diversity, species richness and abundance of individuals. In Refuge B, *Bidens pilosa* and *Nicandra physaloides* showed a strong occurrence of the main parasitoids that attack *S. frugiperda*, as well as some of the main predators. Many predators like Coccinellidae and Anthocoridae seem to be highly abundant in *B. pilosa* [27], which also has bracteal extrafloral nectaries. More than 3800 species of angiosperms have extrafloral nectaries [26], which are considered as an indirect defence against herbivory, since they attract natural enemies of herbivores, especially ants [28]. Adults of many insects, especially Hymenoptera, need nectar to complete their diet and assist with the energy requirements for flight [24,29,30]. Additionally, it has been shown that extrafloral nectaries may induce higher longevity and parasitization rates [5,31,32], and they are available for longer periods of time than floral nectar. They may also feed on honeydew from non-host aphids, but its effects over fecundity or longevity are not as good as when they feed on nectar, although it may be used as a suitable food source when flowering plants are absent [33]. Therefore, extrafloral nectaries are highly relevant to enhance natural control by increasing the specialized natural enemies’ populations. Actually, Hymenoptera parasitoids seem to have an innate attraction to extrafloral nectaries [32], and they are the non-ant insects most frequently reported as visitors of extrafloral nectaries [34]. However, floral sources need to be close to the crop in order to limit the time cost of travelling from plant resources to hosts [6].

The number of predators was not significantly related to the presence of extrafloral nectaries in Refuge C, where *Phaseolus vulgaris*, that has extrafloral nectaries in the stipules instead of the bracts, was dominant. *P. vulgaris* is the only species amongst the five plants in Refuge C that had this attribute, and these data reinforce our observation that predators and parasitoids more frequently visit reproductive organ extrafloral nectaries. Bracteal extrafloral nectaries are more suitable for the natural enemies than nectaries present in other plant parts, such as leaves, since they produce more nectar and this production is independent from foliar damage [35]. Furthermore, several authors have claimed that foliar extrafloral nectaries are a defence strategy developed especially to attract ants [34]. Although many other insects have been reported to use these sources, the presence of ants seems to cause other non-ant predators to refrain from visiting those plants, even if they have extrafloral nectaries [36]. Additionally, in some cases, extrafloral nectaries may attract herbivores, as shown in *Phaseolus lunatus* [34], so that, if it also happens in *P. vulgaris*, the interguild competition may reduce access of natural enemies to these sources.

Abundance and richness of both predators and parasitoids was significantly related to a high foliage density in Refuge A which may, in turn, be related to the highest presence of herbivores in these plants, since abundance of these two functional groups was correlated to the abundance and richness of herbivores, as reported by others [37]. Plants that are being damaged by herbivores emit several chemicals, so-called herbivore-induced plant volatiles (HIPVs) that tell predators and parasitoids that their hosts are present [38,39,40]. However, natural enemy foraging behaviour, especially in parasitoids [38], may be affected by the presence of non-host herbivores. The presence of non-hosts, despite parasitoid preference for plants with a combination of hosts and non-hosts, can reduce parasitism rates, depending on the host, because of the higher search times [41,42]. We were not able to identify if neighbouring plant species or allelopathic effects amongst plant species affected the potential of parasitoids in finding hosts [43], and these points should be addressed in future research. Additionally, several plant traits not measured in this survey may also affect herbivory, such as foliar toughness [44], other structural features [45] or even the plant chemistry and nutritional value, which, in turn, may also be affecting the parasitoid or predator performance [46,47]. Significantly more predators were found in herbaceous than shrubby plants in all refuges, which is likely related to a higher abundance of herbivores in these species because structural or morphological plant traits can act as a primary defence against herbivory [48,49]. However, parasitoids seemed to prefer shrubs over woody plants in Refuge B, and shrubs over herbs when considering parasitoids richness, which may be an artefact because only one plant species had those traits and no significant results were found in the other refuges; this relationship should be more deeply studied.

No parasitoid species were collected on maize crops in part because our sampling was biased toward collecting adults and sweep netting was not feasible in the maize plot because of the architecture and toughness of the plants. Another reason may be the above-mentioned application of pesticides for control of *S. frugiperda* and these chemicals are more harmful to parasitoids than predators [50]. The resulting reduction of *S. frugiperda* populations and other insect pests limits the availability of hosts and survival of parasitoids. Despite these biases, several parasitoids that attack *S. frugiperda* were found in the refuges: *Eucelatoria* sp. 1, *Chelonus insularis* and *Apanteles* sp. 1, all of which were mainly present in Refuges A and B (especially in *F. vulgare*, *N. physaloides*, and *B. pilosa*). Furthermore, the occurrence of *Spodoptera eridania* in several plant species, but mostly on *N. physaloides* (Refuge B), may promote natural control of *S. frugiperda*. It does not cause great damage on maize (actually, it was absent in the maize samples in the present survey), but it is an alternative host for the parasitoids *Eucelatoria* sp. 1, *C. insularis*, and *Campoletis flavicinta* [51]. Indeed, we reared several parasitoids belonging to these species from caterpillars of *S. eridania* collected in *N. physaloides* [52].

Another very useful parasitoid species was the Aphidiinae (Braconidae) *Praon volucre*, which has been reported as a parasitoid of *R. maidis* [53], and was only present in *H. annus* (refuge B).

Refuges A and B had the highest predator richness, including the main predators collected in maize. A higher predator diversity enhances herbivore control [54]. Although *Condylostylus similis* was the most abundant predator in maize, *Chrysoperla externa* [51], given the numbers of Chrysopidae nymphs we found in maize and Coccinellidae beetles [27], may be more abundant than reported by this study. In spite of being less abundant, the highly-voracious aphid predators *Allograpta exotica* and *Orius insidiosus* also need to be taken into consideration. *Rhopalosiphum maidis*, the only aphid present in the maize crop, was absent in the late samples. Therefore, to maintain a stable population of insects that feed on aphids, alternative hosts, one of which was only present on *H. annus* (Refuge B), are important to maintain in a refuge.

## 5. Conclusions

This study illustrates the value of planting several carefully selected plant species for enhancing natural enemies for control of maize pests. Considering the limitations of this, or any, study including the location of refuge patches on the maize plots treated with pesticides, one of the most interesting results is that the presence of bracteal extrafloral nectaries seems critical for the presence of parasitoid natural enemies of maize pests. Despite promising results reporting the role of extrafloral nectaries on herbivore suppression (or, at least, on attracting predators and parasitoids), these plant resources are not widely used. Indeed, it appears that there is still a general belief (and a consequent fear) that nectaries also attract herbivores [34]. However, the evidence we present indicates a strong relationship between these particular plant resources and the presence of predators and parasitoids in refuge plantings. The next step will be to assess the impact of selected plant species on herbivore suppression in maize crops, without the interference of pesticides or other methodological bias.

## Figures and Tables

**Table 1:** Species selected for the refuge patches, including their plant features. Families are indicated between brackets: Apiaceae (Ap), Asteraceae (As), Fabaceae (F), Lamiaceae (L), Malvaceae (M), Poaceae (P), and Solanaceae (S).

Refuge	Species	Type	Life Form	Foliage Density	Extrafloral Nectaries
A	*Gossypium barbadense* (M)	Cultivated	Herb	High	Yes (bracts)
	*Aster* sp (As)	Ornamental	Herb	Medium	No
	*Foeniculum vulgare* (Ap)	Aromatic	Herb	Medium	Yes (bracts)
	*Coriandrum sativum* (Ap)	Aromatic	Herb	Medium	No
	*Lavandula officinalis* (L)	Aromatic	Herb	Medium	No
B	*Helianthus annus* (As)	Cultivated	Herb	Medium	Yes (bracts)
	*Nicandra physaloides* (S)	Weed	Shrub	High	No
	*Salvia officinalis* (L)	Aromatic	Woody	Medium	No
	*Bidens pilosa* (As)	Weed	Shrub	Medium	Yes (stem, nodes, bracts)
	*Artemisia absinthium* (As)	Weed	Shrub	Medium	No
C	*Malva parviflora* (M)	Weed	Herb	High	No
	*Rosmarinus officinalis* (L)	Aromatic	Shrub	Medium	No
	*Phaseolus vulgaris* (F)	Cultivated	Herb	Medium	Yes (stipules)
	*Galinsoga parviflora* (As)	Weed	Herb	Medium	No
	*Sorghum halepense* (P)	Cultivated	Herb	Medium	No

**Table 2:** Total number of individuals (N) and species (S) belonging to each functional group collected in each plant species in each refuge, including maize crops.

		Herbivores		Parasitoids		Predators		Total	
Refuge	Plant Species	N	S	N	S	N	S	N	S
A	*Aster* sp.	22	9	41	27	142	15	214	54
	*Coriandrum sativum*	72	4	75	31	260	17	407	52
	*Foeniculum vulgare*	222	5	276	54	901	30	1403	90
	*Gossypium barbadense*	671	15	229	52	715	27	1627	97
	*Lavandula officinalis*	6	2	54	26	117	4	183	34
Total Refuge A		993	21	675	71	2135	41	3803	133
B	*Artemisia absinthium*	1	1	25	17	44	2	70	20
	*Bidens pilosa*	97	8	390	60	149	23	687	94
	*Helianthus annus*	336	13	182	13	429	21	957	50
	*Nicandra physaloides*	499	10	177	33	165	14	856	59
	*Salvia officinalis*	11	5	25	19	76	9	118	34
Total Refuge B		944	17	799	72	863	31	2606	120
C	*Galinsoga parviflora*	23	9	104	26	189	11	316	46
	*Malva parviflora*	376	11	145	41	348	21	878	77
	*Phaseolus vulgaris*	159	7	15	9	90	3	265	20
	*Rosmarinus officinalis*	24	1	17	14	15	4	56	19
	*Sorghum halepense*	43	7	148	24	240	8	431	39
Total Refuge C		625	19	429	59	882	27	1936	105
	Maize	2138	15	207	5	1769	18	4144	38
Total		4700	38	2110	89	5649	52	12,459	179

**Table 3:** Number of species observed (S obs) and predicted by the two estimators (S ICE, S Jack1) in every plant species, including maize crops, and every refuge. Sampling effectiveness is presented as the proportion of observed species to the estimated species.

					Effectiveness	
Refuge	Plant Species	S obs	S ICE	S Jack1	ICE	Jack 1
A	*Aster* sp.	61	132	97	46.26%	62.96%
	*Coriandrum sativum*	60	98	86	61.21%	69.41%
	*Foeniculum vulgare*	99	121	123	81.71%	80.74%
	*Gossypium barbadense*	106	142	142	74.82%	74.71%
	*Lavandula officinalis*	36	55	54	65.71%	66.82%
Total Refuge A		133	150	159	88.93%	83.52%
B	*Artemisia absinthium*	22	74	36	29.61%	61.23%
	*Bidens pilosa*	100	123	128	81.06%	77.98%
	*Helianthus annus*	57	89	80	64.15%	70.86%
	*Nicandra physaloides*	68	112	100	60.92%	70.15%
	*Salvia officinalis*	36	116	58	31.05%	61.76%
Total Refuge B		120	132	140	90.93%	85.48%
C	*Galinsoga parviflora*	52	99	77	52.64%	67.17%
	*Malva parviflora*	83	123	114	67.57%	72.70%
	*Phaseolus vulgaris*	23	36	31	63.33%	73.46%
	*Rosmarinus officinalis*	20	64	32	31.19%	62.25%
	*Sorghum halepense*	42	65	60	64.90%	70.07%
Total Refuge C		105	142	138	73.94%	76.05%
	Maize	40	46	48	86.58%	34.21%

**Table 4:** Results obtained from the Kruskal-Wallis tests between the parasitoid and predator assemblages’ attributes and the plant attributes, calculated separately for every refuge. The extrafloral nectaries in species from refuges A and B are present in bracts, whilst those present in Refuge C are in stipules. Tests for life form could not be done for Refuge A, since all plant species there were herbaceous. Significance level is shown between brackets. Values shown in bold were statistically significant (*p* < 0.05).

Plant attributes	Functional group	Insect attributes	Refuge A	Refuge B	Refuge C
Extrafloral nectaries	Parasitoids	Abundance	**25.96 (0.000)**	**9.38 (0.002)**	**8.98 (0.003)**
		Richness	**23.78 (0.000)**	**4.82 (0.028)**	**8.06 (0.005)**
	Predators	Abundance	**75.72 (0.000)**	**11.05 (0.001)**	0.38 (0.539)
		Richness	**83.30 (0.000)**	**24.98 (0.000)**	0.22 (0.639)
Foliage density	Parasitoids	Abundance	**5.97 (0.015)**	2.33 (0.127)	0.28 (0.594)
		Richness	**5.11 (0.024)**	0.63 (0.427)	0.63 (0.427)
	Predators	Abundance	**29.29 (0.000)**	1.22 (0.269)	**10.71 (0.001)**
		Richness	**26.56 (0.000)**	0.03 (0.874)	**6.18 (0.013)**
Life form	Parasitoids	Abundance	**-**	**13.34 (0.001)**	1.06 (0.304)
		Richness	**-**	**16.19 (0.000)**	0.43 (0.514)
	Predators	Abundance	**-**	**23.71 (0.000)**	**34.78 (0.000)**
		Richness	**-**	**24.97 (0.000)**	**28.98 (0.000)**

## Supplementary Materials

The following is available online at https://www.mdpi.com/2075-4450/8/3/71/s1. Table S1: Abundance of all insect species in every plant species.
