# Supplementary material for: Do Refuge Plants Favour Natural Pest Control in Maize Crops?
Source: Insects. 2017 Jul 18;8(3):71. doi: 10.3390/insects8030071 (PMC5620691; doi:10.3390/insects8030071)
Supplement: Supplementary file 1 [file insects-08-00071-s001.pdf]

Supplementary Materials

Table S1. Abundances of all insect species in every plant species, including maize. All insect species are sorted by functional group, families, and orders. As: *Aster* sp.; Csa: *Coriandrum sativum*; Fvu: *Foeniculum vulgare*; Gba: *Gossypium barbadense*; Lof: *Lavandula officinalis*; Aab: *Artemisia absinthium*; Bpi: *Bidens pilosa*; Han: *Helianthus annus*; Nph: *Nicandra physaloides*; Sof: *Salvia officinalis*; Gpa: *Galinsoga parviflora*; Mpa: *Malva parviflora*; Pvu: *Phaseolus vulgaris*; Rof: *Rosmarinus officinalis*; Sha: *Sorghum halepense*; Ma: Maize.

	Refuge A					Refuge B					Refuge C					
	As	Csa	Fvu	Gba	Lof	Aab	Bpi	Han	Nph	Sof	Gpa	Mpa	Pvu	Rof	Sha	Ma
**HERBIVORES**																
COLEOPTERA																
Fam. Chrysomellidae																
Diabrotica viridula (Fabricius, 1801)				8			2	7		1						436
Epitrix sp. 1								3	174							
Fam. Curculionidae																
Anthonomus vestitus Boheman, 1859				8												
Fam. Nitidulidae																
Carpophilos sp. 1																75
DIPTERA																
Fam. Agromyzidae																
Liriomyza huidobrensis (Blanchard, 1926)	1			7	4	1	13	19	15	4	5	77	24		19	
Fam. Ulidiidae																
Euxesta sp. 1				34								1			5	71
HEMIPTERA																
Fam. Aleyrodidae																
Bemisia tabaci (Gennadius, 1889)				265			7	54	3			111	52			
Fam. Aphididae																
Aphis gossypii Glover, 1877		54	212	142				64	24		2	38	16			
Aphididae sp. 1								47								
Rhopalosiphum maidis (Fitch, 1856)																128
Fam. Cicadellidae																
Dalbulus maidis Delong & Wolcott, 1923		4						1					2			102

Empoasca kraemeri Ross & Moore, 1957	3			123		9	96	6		8	1	54		4	38
Fam. Delphacidae															
Peregrinus maidis (Ashmead, 1890)	3			23			37	1	2	1		7			45
Fam. Miridae															
Miridae sp. 1	3		6	13		17			2		79				
Sthenaridea carmelitana (Carvalho, 1948)															397
Fam. Ortheziidae															
Orthezia sp. 1													24		
Fam. Pentatomidae															
Euschistus sp. 1	3		2	2				2							
Fam. Pyrrhocoridae															
Dysdercus peruvianus Guerin, 1831				25											
LEPIDOPTERA															
Fam. Gelechiidae															
Gelechiidae sp. 1	2	13	1		2	1								5	
Pectinophora gossypiella (Saunders 1844)				1											
Fam. Hesperiidae															
Hesperidae sp. 1	4	1				2		2	2	1					
Urbanus sp. 1			1							1	1				
Fam. Noctuidae															
Agrotis sp. 1															2
Anomis texana (Riley, 1885)				5											
Heliothis virescens (Fabricius, 1777)				9			1								5
Pseudoplusia includens (Walker, 1857)							3	14			25	4			
Spodoptera eridania (Cramer, 1782)						46	2	258		3	18			1	
Spodoptera frugiperda (Smith, 1797)															144
Fam. Pieridae															
Leptophobia aripa (Boisduval, 1836)	1									1					
Fam. Pyralidae															

Diatraea saccharalis (Fabricius, 1794)														2
Elasmopalpus lignosellus (Zeller, 1848)														3
Marasmia trapezalis (Guenée, 1854)													6	
Pyralidae sp. 1														1
Tallula atramentalis (Lederer, 1863)				6						1				
Fam. Sphingidae														
Sphingide sp. 1											22		3	
ORTHOPTERA														
Fam. Acrididae														
Acrididae sp. 1											3			
THYSANOPTERA														
Fam. Thripidae														
Frankliniella williamsi Hood, 1915														689
Thripidae sp. 1	2						2							
**PARASITOIDS**														
DIPTERA														
Fam. Bombyliidae														
Villa sp.1			2											
Fam. Tachinidae														
Tachinidae sp. 1														1
Eucelatoria sp. 1								64						
Whintemia sp. 1														3
Winthemia sp. 2				3										
Winthemia reliqua Cortés & Campos, 1971														3
HYMENOPTERA														
Fam. Bethylidae														
Epyrinae sp. 1		2		1		2	1		1			1	1	
Epyrinae sp. 2		1				1		1		1		1		
Epyrinae sp. 3	1		1	2	1									
Fam. Braconidae														
Aleiodes sp. 1											1			

Alphomelon sp. 1						1	2									
Alysiinae sp. 1														1		
Apanteles sp. 1	2	1	22	5	7		53	2	3	1	2	4	1	1		
Aphidiinae sp. 1		3	4	3												
Aphidiinae sp. 2			5	2	2			2		2	2	2				
Aphidiinae sp. 3																193
Braconinae sp. 1	1	1	3	1			1				3					
Braconinae sp. 2		1	3	3	1	1	4			1		1				
Braconinae sp. 3	2	1	11	3			9		2						1	
Braconinae sp. 4		4	3	3	3		7			1		1				
Cheloninae sp. 1	2	1	1	3	1		13	1		2						
Chelonus insularis Cresson, 1865	1		10	2			16		22		2				3	
Euphorinae sp. 1				1												
Heliconinae sp. 1							1									
Iconella sp. 1		3	2				1									
Leiophron sp. 1		3	10	20	2		8	1				4			13	
Opiinae sp. 1	1			1			2					4				
Opiinae sp. 2	2		1	2			4									
Opiinae sp. 3				2			11					12				
Praon volucre (Haliday, 1833)								162								
Rogadinae sp. 1						1				1						
Fam. Chalcididae																
Brachymeria sp. 1				3			4									
Brachymeria sp. 2	1		2				4		2			3				
Chalcidini sp. 1							12		1		1					
Spilochalcis sp. 1							10				1					
Fam. Diapriidae																
Diapriinae sp. 1					1		3					1				
Fam. Encyrtidae																
Encyrtidae sp. 1		3	10	18	9		3						1			
Encyrtidae sp. 2	2	2	2	4	1											
Encyrtidae sp. 3			2	23												
Encyrtidae sp. 4	2	1	6	5				2	1			1				
Encyrtidae sp. 5			3	2		1	2		4			1			2	

Fam. Eucolidae																
Eucolidae sp. 1	1	3	17	22	1		11			1	5	9	2	2	3	
Eucolidae sp. 2	1	2	1	1			3					1				
Eucolidae sp. 3			2	1	3		1		1		1	4			1	
Eucolidae sp. 4	1	1	4		2		1		2			2				
Fam. Eulophidae																
Eulophidae sp. 1	2			4			3		1	1		3				
Eulophidae sp. 2	1	3	2	2	3		2					3		1		
Eulophidae sp. 3			3	1	1	1			2			5			6	
Eulophidae sp. 4	1	7	24	9	1	5	14	1	3	1	3	11		2	2	
Eulophidae sp. 5		1					2			3	1	3		1	54	
Eulophidae sp. 6		1	5	9	1	3			4		1	17	1		27	
Fam. Eupelmidae																
Eupelminae sp. 1							1				1	1			9	
Fam. Eurytomidae																
Eurytomidae sp. 1			4	4												
Eurytomidae sp. 2		1	4				1					1				
Eurytomidae sp. 3			2				2	1			1					
Fam. Figitidae																
Figitinae sp. 1		1														
Fam. Ichneumonidae																
Anomaloninae sp. 1				1											1	
Campoletis flavicinta (Ashmead, 1890)																7
Campopleginae sp. 1			2				2		1			2				
Campopleginae sp. 2		1	2				2			1						
Cremastinae sp. 1				1		1	4					1				
Cremastinae sp. 2			2	2	1	1	4					1				
Cryptinae sp. 1			1				13	1				1				
Diplazon laetatorios (Fabricius, 1781)	2		4		1		1		1						1	
Orthocentrinae sp. 1							2									
Pimpla sp. 1							2									
Fam. Mutilidae																

Timulla sp. 1							1								
Fam. Mymaridae															
Mymaridae sp. 1	1		1			1	3			1					
Mymaridae sp. 2					1						1	1			
Fam. Perilampidae															
Perilampidae sp. 1			3	9	1		25		5			2			
Perilampidae sp. 2	1		4	1					3			1	2		
Fam. Platigastridae															
Platigastridae sp. 1			1	1			1								
Fam. Pteromalidae															
Pteromalidae sp. 1	1		1				4				1				1
Pteromalidae sp. 2	2	7	17	7			8	6	6	1		1		1	3
Pteromalidae sp. 3		1	2	2			21		3	2	2	2			10
Pteromalidae sp. 4		9	14	8	2	1	11	1	3		6	6		1	2
Pteromalidae sp. 5	2		2	2			12		1		1				1
Pteromalidae sp. 6			6	1		1	12				1			1	
Pteromalidae sp. 7			3	4			5		1			2	1		
Pteromalidae sp. 8	1		2	3	3		12	1	5	1		4		1	2
Fam. Scelionidae															
Scelioninae sp. 1	1		5	4	2		2		1	2	2	1			
Scelioninae sp. 2		1	4	4	1	2	4		2		1	2	3	1	1
Scelioninae sp. 3	4	1	1	4	2	2	3				1	4	3	2	
Scelioninae sp. 4				1		1	1		2	1	1	3			
Teleasinae sp. 1	2	2	3	1	1		9		3	1	2		1		
Fam. Scoliidae															
Campsomeris sp. 1		6	24			1	12		22		60	16			2
Fam. Tiphidae															
Anthoboscinae sp. 1				6					1						1
Fam. Torymidae															
Podagrion sp. 1			1						3						1
Podagrion sp. 2				2					1						
**PREDATORS**															
COLEOPTERA															
Fam. Carabidae															

Megacephala carolina chilensis (Laporte, 1834)	1						10			4						
Pterostichus sp. 1				2			7			4		12				
Pterostichus sp. 2																2
Fam. Coccinellidae																
Coccinellidae sp. 1	7	48	218	10				23				39				
Coccinellidae sp. 2																7
Cryptolaemus montrouzieri (Mulsant, 1853)		1		4			1	2								
Cycloneda sanguinea (Linnaeus, 1763)		1	3	2			2	11				4				
Eriopis connexa connexa (Germar, 1824)			1	1			6	8				3				
Harmonia sp. 1	2	46	190	22				6	5			34				
Harmonia sp. 2																10
Hippodamia convergens (Guerin-Meneville, 1842)	1	5	17		3		3	12	4			16		1	2	5
Scymnus sp. 1		1	28	1				41				4				
Zagreus heasticta (Crotch, 1874)				1			2					1				
DIPTERA																
Fam. Asilidae																
Lochmorhynchus albicans (Carrera & Andretta, 1953)																1
Fam. Dolichopodidae																
Condylostylus sp. 1	1			13			8	2	1			2				
Condylostylus similis (Aldrich, 1901)	37	11	6	114	3	5	38	64	79	5	11	140	33	2	9	76
Fam. Syrphidae																
Allograpta exotica (Wiedemann, 1830)	60	92	135				20		10	1	31	11			122	29
Allograpta piurana Shannon, 1927	3	25	43				3				3				14	
Syrphidae sp. 1																84
Syrphidae sp. 2		2	3				1	15				9			3	

Pseudodorus clavatus (Fabricius, 1794)	2	3	25			3	1	1		11	3		4	
Syrphus shorae Fluke, 1950	2	2	5			1	2						8	
HEMIPTERA														
Fam. Anthocoridae														
Orius insidiosus (Say, 1832)	4			120			24	16		17	15	15		57
Fam. Berytidae														
Metacanthus tenellus Stål, 1859		3	3	131		5	67	10	9	6	25			9
Fam. Lygaeidae														
Geocoris punctipes (Say, 1832)			1	15		2	11		3	1	2			7
Fam. Miridae														
Hyalochloria denticornis Tsai-Yu-Hsiao, 1945			1	10			2							
Rhinacloa aricana Carvalho, 1948				1							1			
Fam. Nabidae														
Nabis punctipennis Blanchard, 1852	2		3	30		3		8	1	2				15
Fam. Pentatomidae														
Podisus sp. 1		2	4	5				1			2			
HYMENOPTERA														
Crabronidae														
Bembicinae sp. 1	1													
Cercerini sp. 1			2	3		1	1	1						
Cercerini sp. 2						1								
Rubrica surinamensis (De Geer, 1778)				1										
Trypoxylon sp. 1			1	3		2	1		1	1				
Fam. Pompilidae														
Pompilidae sp. 1			1	2										
Pompilidae sp. 2	1		2											
Pompilidae sp. 3			1			2								
Pompilidae sp. 4			1											
Tachypompilus sp. 1					1									
Fam. Sphecidae														
Ampulex sp. 1			3											

Sceliphron caementarium (Drury, 1773)			28	2												
Sphecidae sp. 1		1														
Sphecidae sp. 2														1		
Fam. Vespidae																
Eumenes canaliculatus Saussure, 1852		1	4	1												
Monobia incarum Bequard, 1940			47	11					1		25					
NEUROPTERA																
Fam. Chrysopidae																
Ceraeochrysa cincta (Schneider, 1851)			4	1				3	1			1				6
Chrysoperla externa (Hagen, 1861)	18	16	118	208	110	39	27	132	27	48	81	23	42	11	78	37
Chrysopidae egg																1232
Chrysopidae nymph																103
Fam. Hemerobidae																
Hemerobius sp. 1																89
Fam. Myrmeleontidae																
Myrmelion sp. 1			3	1			1	1				1				
